# L-Proline protects human sperm from heat stress-induced oxidative damage

**DOI:** 10.1007/s00726-026-03524-2

**Published:** 2026-06-12

**Authors:** Mojtaba Moradi, Mohsen Shayestehyekta, Amir Hossein Hashemian, Azita Faramarzi

**Affiliations:** 1 Fertility and Infertility Research Center, Health Technology Institute, Kermanshah University of Medical Sciences, Kermanshah, Iran; 2 Department of Biostatistics, School of Health, Kermanshah University of Medical Sciences, Kermanshah, Iran; 3 Research Center for Environmental Determinants of Health (RCEDH), Health Institute, Kermanshah University of Medical Sciences, Kermanshah, Iran; 4 Department of Anatomical Sciences, Medical School, Kermanshah University of Medical Sciences, Kermanshah, Iran

**Keywords:** L-proline, Human sperm, Heat stress, Oxidative stress, Antioxidant

## Abstract

L-proline, an amino acid with antioxidant properties, has demonstrated significant potential in counteracting oxidative stress and preserving sperm quality. This study evaluated the protective effects of L-proline against heat stress-induced damage during sperm preparation incubation, a critical step in assisted reproductive technologies (ART). Semen samples from 30 normozoospermic men were divided into two groups: a control group and a treatment group supplemented with 2 mmol/L L-proline. Following initial incubation at 37 °C for 30 min, samples were further subdivided and subjected to controlled thermal exposures at 25 °C, 37 °C, and 42 °C for both short-term (3 h) and long-term (24 h) incubation periods, simulating physiological and heat-stress conditions. Sperm parameters, including motility, viability, morphology, and biochemical markers of oxidative stress, including catalase (CAT), superoxide dismutase (SOD), total antioxidant capacity (TAC), malondialdehyde (MDA), and nitric oxide (NO), were comprehensively assessed. Results indicated that heat stress exposure significantly impaired sperm function and disrupted redox homeostasis. Supplementation with 2 mmol/L L-proline significantly preserved sperm motility and viability during heat stress conditions compared to the control group (*p* < 0.05). However, no significant protective effect on sperm morphology was observed. Additionally, L-proline enhanced antioxidant defenses by improving CAT, SOD, and TAC levels while concurrently modulating MDA and NO levels under heat stress conditions (*p* < 0.05). In conclusion, these findings highlight the potential of L-proline as a protective supplement to ameliorate the detrimental effects of heat stress on human sperm quality and oxidative status, suggesting that incorporating L-proline into sperm preparation protocols may offer a promising strategy to enhance clinical outcomes.

## Introduction

Male infertility is a critical global health concern, affecting approximately 50 million reproductive-aged couples worldwide (Agarwal et al. 2021; Eisenberg et al. 2023). Over recent decades, a notable decline in sperm quality, encompassing sperm concentration, volume, and motility, has emerged as a primary contributor to male infertility (Agarwal et al. 2021; Eisenberg et al. 2023). While genetic defects account for some of these changes, lifestyle factors, psychological stress, exposure to exogenous chemicals, and elevated temperatures are also key contributors (Sharma et al. 2013; Virolainen et al. 2023). These challenges have driven advancements in assisted reproductive technologies (ART), with a particular focus on identifying compounds that can enhance sperm functionality and improve success rates in infertile and sub-fertile men (Jain and Singh 2022).

Heat stress (HS) has garnered growing attention as a significant factor influencing male reproductive health (Shahat et al. 2020). Elevated testicular temperatures, often linked to lifestyle habits, occupational exposures, and the broader impact of climate change, have been shown to induce HS, which disrupts spermatogenesis (Cao et al. 2023; Shahat et al. 2020). Heat stress triggers the overproduction of reactive oxygen species (ROS), resulting in testicular tissue damage, reduced sperm count, motility, and morphology, as well as DNA fragmentation and oxidative stress in spermatozoa (Hamilton et al. 2016; Wang et al. 2025). As global temperatures rise, the implications of heat stress on male fertility represent an increasingly pressing concern (Boni 2019; Walsh et al. 2019).

The process of sperm incubation, integral to ART cycles, involves maintaining sperm in a controlled environment, typically at 37 °C, to optimize its readiness for use (Group 2020; Henkel and Schill 2003). The incubation period, ideally under 1.5 h, is critical for preserving sperm motility and viability, as prolonged exposure may impair these parameters (Jain and Singh 2022). Although some studies suggest that extended incubation (up to 24 h) prior to intracytoplasmic sperm injection (ICSI) can enhance pregnancy rates, prolonged incubation at 37 °C with 5% CO2 has been shown to negatively affect sperm DNA integrity (Matsuura et al. 2010; Moradi et al. 2022b). In prior research, our team demonstrated that 24-h incubation of human sperm adversely impacts sperm parameters and chromatin quality, highlighting the need for improved incubation strategies (Moradi et al. 2022b). Despite these concerns, prolonged sperm incubation remains a common laboratory practice for various diagnostic and therapeutic purposes in fertility clinics (Moradi et al. 2022b). Given this, evaluating antioxidant supplementation at different doses and incubation intervals appears necessary to counteract oxidative stress (Sikka and Hellstrom 2016). As sperm incubation is a routine and often unavoidable procedure in ART, enriching the sperm culture medium with appropriate antioxidants may offer a promising strategy to preserve sperm quality and genomic stability during laboratory handling (Moradi et al. 2022b).

L-proline, a nonessential amino acid also known as 2-pyrrolidinecarboxylic acid, is synthesized endogenously from amino acids such as L-glutamic acid and is readily available as a dietary supplement (Moradi et al. 2022b; Vettore et al. 2021). It plays a pivotal role in mitigating osmotic and oxidative stress, regulating carbon and nitrogen metabolism, and modulating cell signaling pathways to support cellular survival (Vettore et al. 2021). Recent findings underscore L-proline’s potential to enhance sperm function, with documented improvements in motility, viability, and DNA integrity (Liu et al. 2023; Moradi et al. 2022b). Our previous studies have further established its efficacy in preserving sperm quality during incubation and cryopreservation (Moradi et al. 2022a, b). L-proline appears to exert its effects through multiple mechanisms, including functioning as an osmolyte to stabilize cellular volume, acting as an energy substrate to enhance motility, and exhibiting antioxidant properties that neutralize free radicals and bolster antioxidant defenses (Moradi et al. 2022a).

Hence, this study aimed to evaluate the effects of L-proline supplementation on sperm parameters, including motility, viability, morphology, as well as nitro-oxidative status, under different thermal conditions at 25 °C, 37 °C, and 42 °C during both short-term (3 h) and long-term (24 h) incubation periods. These conditions were designed to simulate physiological and heat stress environments. By elucidating the protective benefits of L-proline, this research seeks to improve sperm incubation protocols and enhance ART outcomes, particularly in cases of thermally compromised sperm quality or suboptimal laboratory and environmental conditions.

## Materials and methods

### Chemicals

The chemicals used in the present study, including L-proline, were purchased from Sigma-Aldrich Co, otherwise noted.

### Semen collection and preparation

This study, conducted from July 2023 to July 2024, received ethical approval from the Institutional Review Board of Kermanshah University of Medical Sciences, Iran (IR.KUMS.MED.REC.1401.174), and was conducted in accordance with the Declaration of Helsinki and related international guidelines for ethical research involving humans. A total of 30 healthy men, all partners of infertile couples, were recruited through the Andrology Division and IVF Clinic at Motazedi Hospital, Kermanshah, Iran. Prior to participation, all subjects provided written informed consent. Semen samples were obtained via masturbation following an abstinence period of 2–7 days. Sperm analysis was carried out according to the World Health Organization (WHO, 2010) guidelines, evaluating semen volume, sperm concentration, total motility, and morphology. Only samples meeting the following inclusion criteria were considered: a minimum semen volume of 1.5 ml, total motility of at least 40%, a sperm concentration of no less than 15 million spermatozoa per ml, and a morphology score of ≥ 4% normal forms. Participants completed a comprehensive questionnaire detailing their occupational background, health status, dietary habits, smoking and alcohol use, and exposure to environmental pollutants. Exclusion criteria encompassed a history of smoking, alcohol consumption, systemic diseases, male accessory gland infections, microorchidism, cryptorchidism, varicocele, or prior use of hormonal therapies, medications, vitamins, or antioxidants. Semen samples were processed using a discontinuous double-layered density gradient centrifugation method in accordance with WHO protocols. This preparation ensured high-quality samples for subsequent analysis (WHO, 2010).

### Study design

**Fig. 1:**
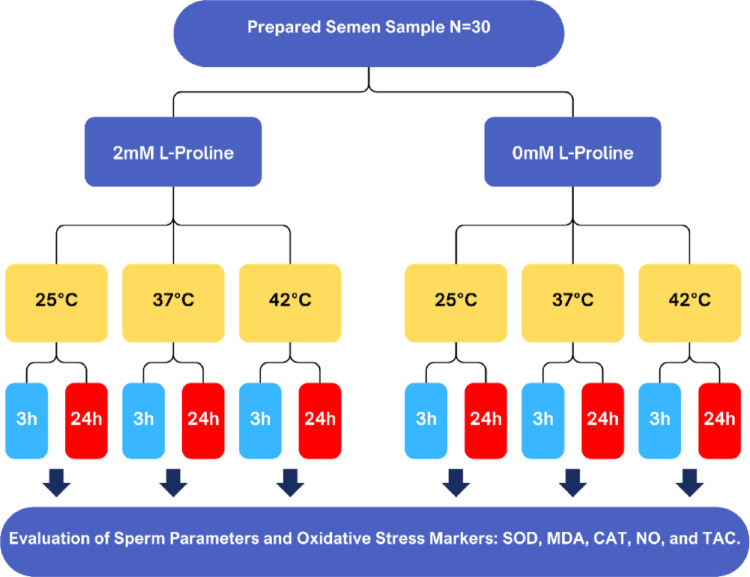
Schematic overview of the experimental design

To prepare the L-proline stock solution, 1.15 mg of L-proline powder was dissolved in 1 ml of Ham’s F-10 medium. The control solution (Ham’s F-10 medium only) and the 2 mmol/L treatment were freshly prepared daily and added to the sperm medium. Each semen sample was divided into two equal aliquots, with one aliquot supplemented with control (Ham’s F-10 medium only) and the other with 2 mmol/L. Both aliquots were pre-incubated at 37 °C (6% CO₂, 95% humidity) for 30 min. Following this initial incubation, each sample was further divided into three portions. These portions were incubated at three distinct temperatures: 25 °C, 37 °C, and 42 °C, with 42 °C serving as a heat stress condition (Gong et al. 2017; Zhao et al. 2021). The samples were maintained at these temperatures for a duration of 3 h and 24 h. Sperm evaluations were conducted at two time points, immediately after the 3-h incubation and again after 24 h of incubation, to assess the effects of the treatments on sperm parameters (Moradi et al. 2022b; Zhao et al. 2021) (Fig. 1).

Semen samples from each donor were equally divided into two main groups: a control group (no antioxidant) and a treatment group supplemented with L-proline (2 mmol/L). Each group was further subdivided into three aliquots and incubated at 25 °C, 37 °C, or 42 °C. Samples were maintained at the designated temperatures for either 3–24 h. Sperm parameters were assessed at both time points to evaluate the effects of heat stress and L-proline treatment.

### Evaluation of sperm parameters

Sperm count was determined using a Neubauer chamber and light microscopy (Olympus Co., Tokyo, Japan). Sperm viability was evaluated using the one-step eosin-nigrosin staining method. A minimum of 200 spermatozoa per sample were analyzed under phase-contrast microscopy at 1000× magnification. Sperm with unstained heads were classified as viable, whereas those with dark pink or red-stained heads were considered non-viable. Sperm motility was analyzed by categorizing 200 spermatozoa into progressive or non-progressive motile groups, following the guidelines established by the World Health Organization (WHO, 2010). The percentage of motile sperm was determined using phase-contrast microscopy at 400× magnification. Sperm morphology was assessed using the Papanicolaou staining method, adhering to WHO (2010) criteria. At least 200 spermatozoa per sample were examined under phase-contrast microscopy at 1000× magnification (Moradi et al. 2025).

### Measurement of lipid peroxidation levels

Malondialdehyde (MDA) concentration was measured using the ZellBio MDA assay kit (ZB-MDA-96 A, ZellBio GmbH, Germany). For the assay, 50 µl of reagent 4 was added to 50 µl of semen sample, followed by 1 µl of the prepared chromogen solution. The mixture was transferred to microtubes, which were then subjected to a boiling water bath followed by an ice bath for 1 h. After incubation, the microtubes were centrifuged at 3000–4000 × g for 10 min. The absorbance of the resulting supernatant was measured at 535 nm using an ELISA microplate reader (Moradi et al. 2022a).

### Total antioxidant capacity measurement

The total antioxidant capacity (TAC) was assessed using the ZellBio TAC assay kit (ZB-TAC-96 A, ZellBio GmbH, Germany). For each sample, 10 µl of sperm sample or reconstituted Trolox at varying concentrations (used as standards) was added to the wells of a microplate. This was followed by the addition of 190 µl of the prepared working chromogen reagent. After a 2-minute incubation at room temperature, the absorbance was measured at 490 nm using a plate ELISA reader (Moradi et al. 2022a).

### Assessment of nitric oxide levels

Nitric oxide (NO) levels in sperm samples were measured using the Griess reagent assay. Sperm samples were diluted to a concentration of 5 × 10⁶ cells/ml in phosphate-buffered saline (PBS, pH 7.4) and aliquoted into sterile tubes. Samples were incubated in substrate buffer containing HEPES (25 mM), NaCl (140 mM), KCl (5.4 mM), CaCl₂ (1 mM), MgCl₂ (1 mM), and NADPH (1.44 mM) with nitrate reductase (20 mU) to convert nitrates to nitrites. The reaction was carried out at room temperature for 1 h and stopped by freezing and thawing the samples. After centrifugation at 1500 × g for 15 min, the supernatant was mixed with an equal volume of Griess reagent (1% sulfanilamide, 0.1% naphthylenediamine dihydrochloride, and 2.5% phosphoric acid). The formation of a colored azo dye was quantified by measuring absorbance at 543 nm using a spectrophotometer. Nitrite concentrations were calculated from a sodium nitrite standard curve and expressed as µmol NO per 10⁶ cells (Balercia et al. 2004).

### Assessment of superoxide dismutase activity

The activity of superoxide dismutase (SOD) was quantified using the spectrophotometric method originally described by Paoletti et al. (1986), which relies on the enzyme’s capacity to inhibit the oxidation of NADH by superoxide radicals. In this assay, the suppression of NADH oxidation serves as an indirect indicator of SOD activity, with a slower rate of NADH oxidation corresponding to higher enzymatic activity. Absorbance was recorded at 340 nm using a UV-visible spectrophotometer, and calculations were based on the molar extinction coefficient of NADH. Results were expressed as units of SOD activity per minute per milligram of protein (U/min/mg protein) (Paoletti et al. 1986).

### Measurement of catalase activity

Catalase (CAT) activity was measured using a colorimetric assay. The reaction mixture (100 µL assay buffer, 20 µL sperm, 30 µL methanol, 20 µL H₂O₂) was incubated at room temperature for 20 min with shaking. The reaction was stopped with 30 µL sodium hydroxide, followed by the addition of 30 µL purpald chromogen and a 10 min incubation. Then, 10 µL potassium periodate was added, and samples were incubated for a final 5 min. Absorbance was measured at 540 nm using a microplate reader (ELx808). Finally, formaldehyde levels were calculated from a standard curve, and results were expressed as nM/min/mL (Johansson and Borg 1988).

### Statistical analysis

Data normality was assessed using the Shapiro-Wilk and Kolmogorov-Smirnov tests. To account for the within-subject, repeated-measures design, data were analyzed using a Linear Mixed-Effects Model (LMM) in R software (lme4 and lmerTest packages). Post-hoc pairwise comparisons between the control and treatment (2 mmol/L) groups at each specific temperature and time point were conducted using estimated marginal means (EMMs) with Tukey adjustment. Results are presented as mean ± standard deviation (SD), and a p-value < 0.05 was considered statistically significant.

## Results

### Effects of L-proline on sperm parameters

#### Effects of L-proline on sperm motility

**Fig. 2:**
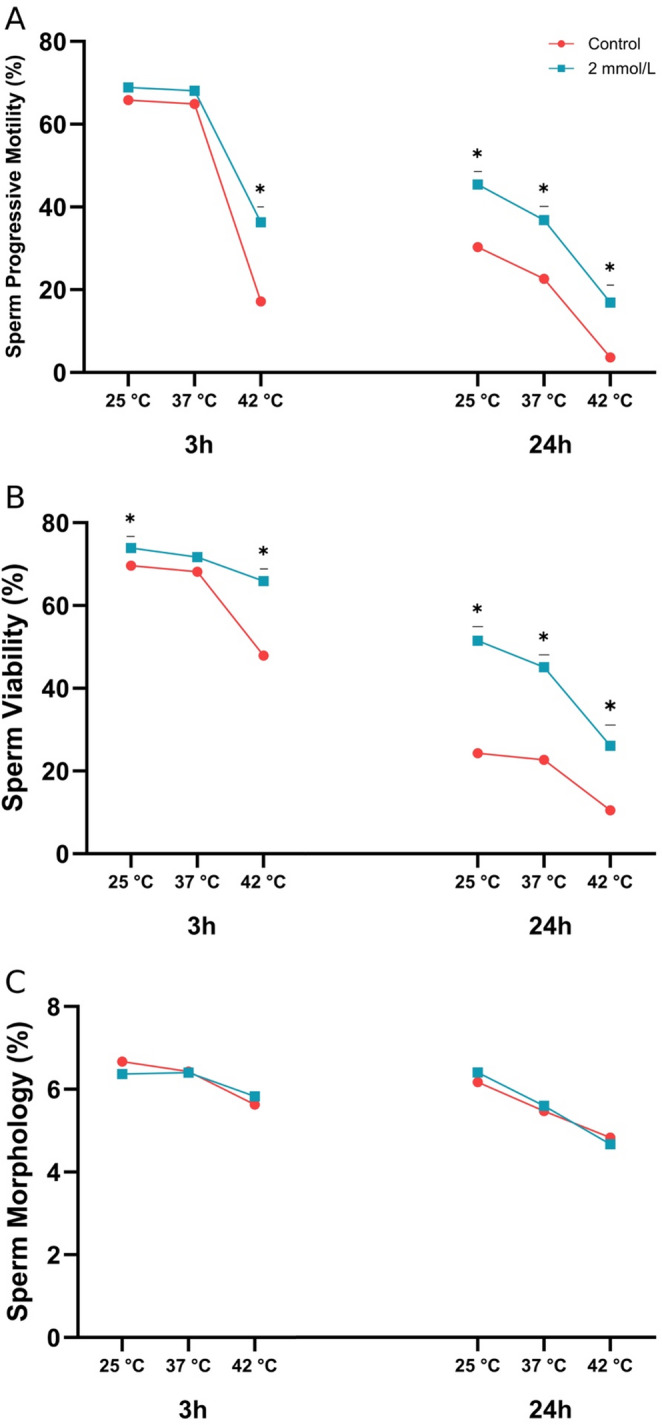
Effects of L-proline supplementation on sperm progressive motility, viability, and morphology during incubation at different temperatures and time points. Data are presented as mean ± SD (*n* = 30). (*) *p* < 0.05 versus control

As expected, sperm motility exhibited a declining trend with increasing temperature and prolonged incubation across all treatment groups. Notably, at 42 °C after 3 h of incubation, the addition of L-proline significantly preserved sperm progressive motility compared to the control group (*p* < 0.05). More importantly, L-proline supplementation effectively maintained sperm motility at various temperatures after 24 h of incubation (*p* < 0.05) (Fig. 2-A). Furthermore, mixed-effects modeling revealed a significant three-way interaction (Treatment × Temperature × times), indicating that the protective efficacy of L-proline on progressive motility is pronounced under compounded thermal and temporal stress (*p* < 0.001).

#### Effects of L-proline on sperm viability

Sperm viability was markedly compromised under heat stress, with a significant decline observed after 24 h of incubation (*p* < 0.05). Importantly, the inclusion of L-proline in the sperm medium substantially preserved sperm viability across different temperatures at both 3 h and 24 h of incubation (*p* < 0.05) (Fig. 2-B).

#### Effects of L-proline on sperm morphology

Heat stress also had a detrimental impact on sperm morphology, particularly after 24 h of incubation (*p* < 0.05) (Fig. 2). However, supplementation with 2 mmol/L L-proline did not significantly protect sperm morphology across different temperature conditions (*p* > 0.05) (Fig. 2-C).

### Effects of L-proline on nitro-oxidative status

#### Effects of L-proline on total antioxidant capacity levels

**Fig. 3:**
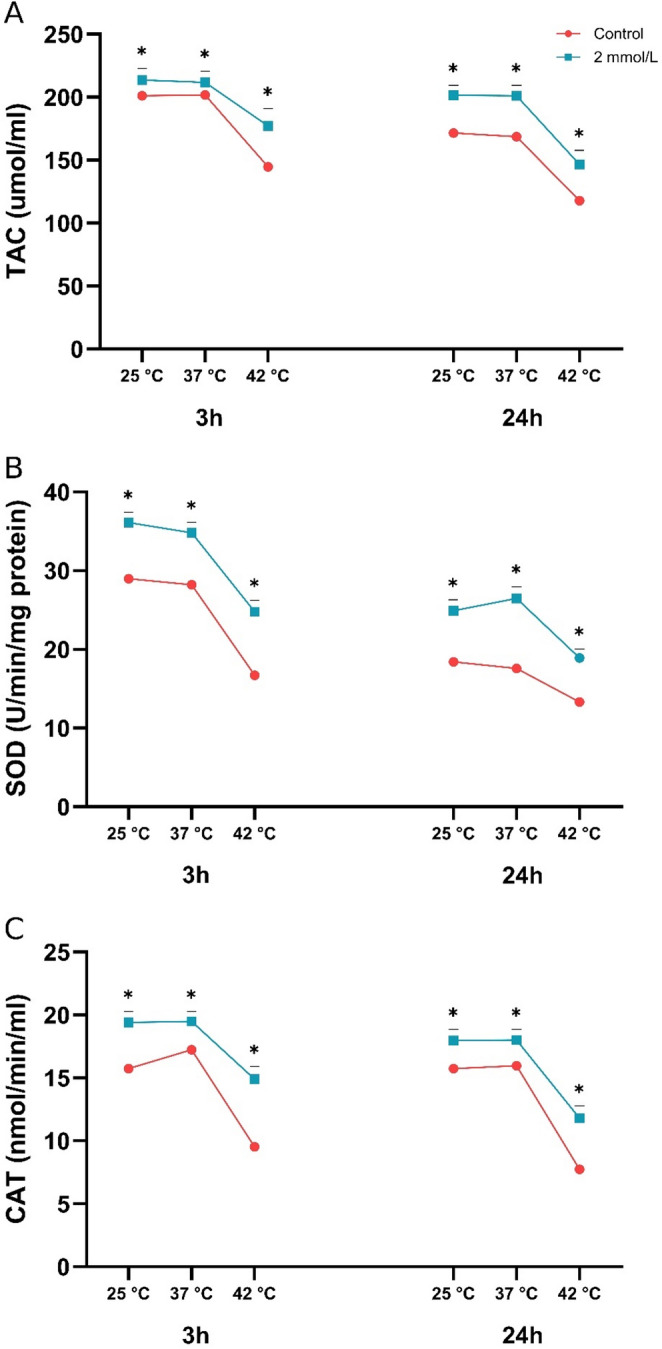
Effects of L-proline supplementation on the TAC (total antioxidant capacity), SOD (superoxide dismutase), and CAT (catalase) levels during incubation at different temperatures and time points. Data are presented as mean ± SD (*n* = 30). (*) *p* < 0.05 versus control

A downward trend in TAC was observed with rising temperatures. However, supplementation with 2 mmol/L L-proline significantly enhanced TAC levels compared to the control group at different temperatures after 3 h of incubation (*p* < 0.05). This effect was further amplified at 24 h, where TAC levels remained significantly higher than in the control group (*p* < 0.05), indicating a time-dependent protective effect of L-proline (Fig. 3-A). A similar significant three-way interaction was observed across the oxidative markers, indicating that L-proline’s enhancement of antioxidant capacity dynamically scales with increasing environmental stress (*p* < 0.001).

#### Effects of L-proline on superoxide dismutase levels

Exposure to heat stress led to a significant reduction in SOD activity at both 3-h and 24-h incubation time points compared to the control group (*p* < 0.05), indicating elevated oxidative stress in human spermatozoa. Notably, supplementation with 2 mmol/L L-proline markedly attenuated this decline, maintaining SOD activity at significantly higher levels under all thermal conditions (*p* < 0.05) (Fig. 3-B).

#### Effects of L-proline on catalase levels

Exposure to elevated temperatures significantly diminished CAT activity in human spermatozoa after both 3 and 24 h of incubation (*p* < 0.05), indicating a disruption in the antioxidant defense system under heat stress conditions. Remarkably, the inclusion of L-proline effectively counteracted this reduction, maintaining CAT activity at levels significantly higher than those observed in the heat-stressed control group (*p* < 0.05). The protective effect of L-proline was particularly pronounced at 42 °C, underscoring its potential role in preserving enzymatic antioxidant capacity under extreme thermal conditions (*p* < 0.05) (Fig. 3-C).

#### Effects of L-proline on lipid peroxidation levels

**Fig. 4:**
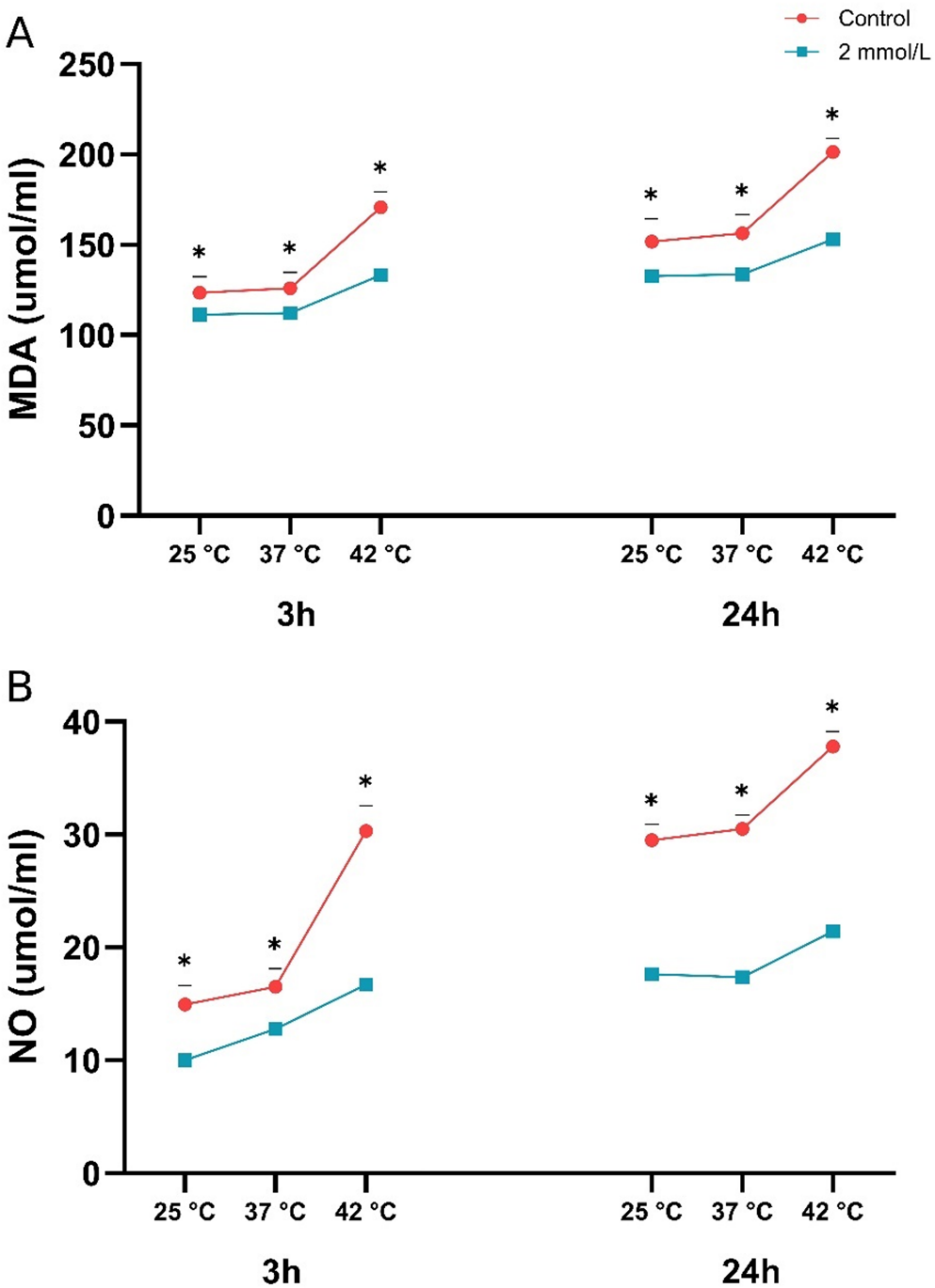
Effects of L-proline supplementation on MDA (malondialdehyde) and NO (nitric oxide) levels during incubation at different temperatures and time points. Data are presented as mean ± SD (*n* = 30). (*) *p* < 0.05 versus control

Figure 4 presents the effects of heat stress on lipid peroxidation, as indicated by elevated MDA levels, which increased significantly with rising temperature, most notably after 24 h of incubation (*p* < 0.05). This rise in MDA reflects enhanced oxidative damage to sperm membrane lipids under thermal stress. However, co-incubation with 2 mmol/L L-proline markedly attenuated this increase, resulting in significantly lower MDA concentrations compared to the heat-stressed control group (*p* < 0.05). The protective effect of L-proline was especially evident at the 24-h time point, suggesting its sustained efficacy in limiting lipid peroxidation during prolonged exposure to elevated temperatures (Fig. 4-A).

#### Effects of L-proline on nitric oxide levels

As shown in Fig. 4, NO levels increased significantly in response to elevated temperatures, particularly at 42 °C during both 3- and 24-h incubation periods (*p* < 0.05), indicating the induction of nitrosative stress under heat stress conditions. This elevation was most prominent following 24 h of exposure, reflecting a time-dependent accumulation of reactive nitrogen species. Importantly, supplementation with L-proline significantly attenuated the heat-induced rise in NO levels (*p* < 0.05), suggesting a protective role in modulating nitrosative stress and preserving the redox balance within the sperm medium (Fig. 4-B).

## Discussion

Recently, the progressive global decline in male fertility has become an increasing concern, with environmental factors such as heat stress, which is exacerbated by climate change, identified as key contributors (Eisenberg et al. 2023; Skakkebæk et al. 2022). L-proline has garnered attention for its multifaceted role in preserving sperm functionality during cryopreservation and long incubation (Abdelnour et al. 2024). Prior studies in both animal and human have highlighted this antioxidant and protective potential (Abdelnour et al. 2024; Moradi et al. 2022b). The present study is the first to provide evidence that supplementing human sperm medium with 2 mmol/L L-proline significantly enhances sperm functional parameters by attenuating nitro-oxidative stress and augmenting antioxidant defenses over both 3- and 24-h incubation periods.

Heat stress is well-documented to compromise sperm motility and viability, as reported in several studies (Nowicka-Bauer and Nixon 2020; Rahman et al. 2018; Zhao et al. 2021). Our findings are consistent with this, demonstrating a marked decline in progressive motility and viability following thermal exposure. Yet, L-proline supplementation effectively preserved sperm viability and motility, indicating its protective efficacy. These results are in agreement with previous reports where antioxidants such as melatonin have been shown to mitigate the detrimental effects of heat stress on sperm motility (Zhao et al. 2021). Similarly, other antioxidants have conferred protection to sperm cells under stress conditions such as cryopreservation and extended storage (Silvestre et al. 2021). The protective role of L-proline is likely attributed to its ability to stabilize mitochondrial function, a critical factor in ATP production and sperm motility (Patriarca et al. 2021). L-proline’s antioxidant properties, particularly its capacity to scavenge ROS, further contribute to its beneficial effects (Abdelnour et al. 2024; Li et al. 2021). Supporting this, previous studies have reported improved motility and viability in ram and bull sperm cells following L-proline supplementation under stress-inducing conditions (Ramazani et al. 2023; Sangeeta et al. 2015). Further, in our earlier study, 2 mmol/L L-proline also maintained sperm function during 24 h of incubation, likely through its role as an osmolyte, preserving cell volume and membrane stability during stress conditions (Bailey et al. 2021; Moradi et al. 2022b).

Interestingly, while L-proline preserved sperm motility and viability after heat exposure, it did not produce a significant improvement in sperm morphology. This may suggest that the morphological defects induced by heat are less a consequence of acute oxidative damage and more the result of irreversible structural injury, such as protein denaturation, membrane collapse, or cytoskeletal disruption, that cannot be readily reversed by a single extracellular antioxidant treatment within 24 h (Capela et al. 2022; Xue et al. 2025). Thus, while L-proline appears effective at mitigating oxidative-stress–mediated functional decline, preventing thermally driven structural damage may require strategies that directly stabilize proteins or membranes or that provide prolonged protection (Kowalczyk 2022).

Oxidative stress, resulting from an imbalance between ROS production and antioxidant defenses, leads to substantial damage to spermatozoa, including DNA fragmentation, reduced motility, and impaired membrane integrity (Hussain et al. 2023). In this study, exposure to heat stress significantly elevated MDA levels, an established marker of lipid peroxidation, highlighting oxidative damage (Demirci-Çekiç et al. 2022). Lipid peroxidation negatively impacts sperm quality by disrupting membrane fluidity and impairing enzyme and ion channel functions critical for motility (Dutta et al. 2021; Wang et al. 2025). Remarkably, L-proline supplementation significantly reduced MDA levels at both incubation points, indicating its strong antioxidative action in mitigating lipid peroxidation and preserving membrane integrity (Abdelnour et al. 2024; Zhang et al. 2022). L-proline’s unique mechanism includes direct ROS scavenging and the formation of electrostatic interactions with phospholipid membranes, thereby reinforcing membrane structure and shielding sperm from oxidative injury (Abdelnour et al. 2024; Zhang et al. 2022). Additionally, its capacity to interact with the phospholipid bilayer contributes to enhanced membrane stability during stress-induced incubation and cryopreservation (Abdelnour et al. 2024; Moradi et al. 2022b).

Nitrosative stress, particularly involving nitric oxide (NO), further contributes to sperm dysfunction under heat stress (Shahat et al. 2020). Although low NO levels are essential for sperm capacitation and motility, elevated NO concentrations impair mitochondrial respiration, motility, and DNA integrity (Nowicka-Bauer and Nixon 2020). Our findings indicate that heat stress significantly increased NO levels, correlating with reduced sperm viability and motility. Notably, L-proline markedly suppressed NO accumulation, especially evident after 24 h of incubation, thereby alleviating nitrosative stress (Costa et al. 2023; Luo et al. 2021). Nguyen et al. (2023) similarly demonstrated that reducing excessive levels of NO can improve sperm quality (Nguyen et al. 2023). Other antioxidants, such as melatonin and butylated hydroxytoluene, have shown similar effects under stress conditions (Pezo et al. 2021; Zhao et al. 2021). Nonetheless, unlike these compounds, L-proline offers the added advantage of acting as an osmolyte, further stabilizing cell volume and enhancing structural resilience under thermal stress (Abdelnour et al. 2024; Zhang et al. 2022).

Total antioxidant capacity (TAC) represents an integrated measure of both enzymatic and non-enzymatic antioxidants and serves as a critical biomarker for assessing oxidative stress in male infertility diagnostics (Barati et al. 2020). Consistently, diminished TAC levels have been linked to reduced sperm quality in infertile men, underscoring the importance of oxidative balance in maintaining sperm functionality (Barati et al. 2020; Saleh et al. 2025). In the present study, heat stress significantly compromised TAC levels after 24 h of incubation, indicating a heightened oxidative burden. Notably, supplementation with 2 mmol/L L-proline effectively preserved TAC levels throughout the incubation period, suggesting a significant antioxidative effect. Additionally, heat-induced stress markedly reduced the activities of SOD and CAT, two key enzymatic antioxidants responsible for neutralizing superoxide radicals and hydrogen peroxide, respectively (Barati et al. 2020). Yet, L-proline supplementation maintained SOD and CAT activity at levels comparable to control conditions, highlighting its potential to support the endogenous antioxidant defense system during prolonged exposure to thermal stress. These results collectively underscore the role of L-proline in fortifying the sperm’s endogenous antioxidant defense systems. Its protective mechanism appears to involve both direct ROS scavenging and the upregulation of cellular antioxidant enzymes (Abdelnour et al. 2024; Ramazani et al. 2023). The observed improvements in motility and viability are linked to the antioxidant and membrane-stabilizing actions of L-proline (Abdelnour et al. 2024). Supporting this, Liu et al. (2021) demonstrated that L-proline enhances the function of both enzymatic and non-enzymatic antioxidant systems, effectively maintaining redox balance and cellular integrity under stress (Liu et al. 2021; Zhang et al. 2022). Our previous research also indicated that 2 mmol/L L-proline supplementation significantly improved TAC levels in human spermatozoa during 24-h incubation (Moradi et al. 2022b).

Despite these promising findings, several limitations must be acknowledged. First, the study population was restricted to normozoospermic men with healthy lifestyle profiles to minimize confounding variables; consequently, these results may not fully generalize to subfertile populations or individuals with underlying pathologies. Second, the study’s in vitro design focuses on laboratory parameters rather than clinical endpoints. While the observed improvements in motility and viability are encouraging, future research is essential to determine if these benefits translate to functional outcomes, such as fertilization capacity and embryo development. Finally, while our findings demonstrate the mitigation of nitro-oxidative stress, the precise molecular signaling pathways underlying L-proline’s protective effects remain to be fully elucidated.

## Conclusion

This study highlights L-proline as a promising antioxidant candidate for protecting human spermatozoa from heat stress-induced oxidative damage. Under in vitro thermal stress, L-proline supplementation was observed to preserve sperm motility and viability by mitigating nitro-oxidative stress and enhancing antioxidant enzyme activity. These findings suggest that L-proline could serve as a beneficial additive to sperm preparation media during ART incubation, particularly where maintaining sperm quality under stress is critical. While primarily relevant to laboratory protocols, these insights may also have broader implications for strategies aimed at counteracting occupational heat stress. Future studies, including clinical trials, are warranted to explore synergistic effects with other antioxidants and to validate these benefits for ART outcomes.

## Data Availability

The datasets used and analyzed during the current study are available from the corresponding author on reasonable request.
